# Protein Extraction Methods Shape Much of the Extracted Proteomes

**DOI:** 10.3389/fpls.2018.00802

**Published:** 2018-06-12

**Authors:** Liangjie Niu, Huayi Yuan, Fangping Gong, Xiaolin Wu, Wei Wang

**Affiliations:** State Key Laboratory of Wheat and Maize Crop Science, Collaborative Innovation Center of Henan Grain Crops, College of Life Sciences, Henan Agricultural University, Zhengzhou, China

**Keywords:** plant proteome, protein extraction, missing proteins, comparative proteomics, organelle subproteomics, single-cell level proteomics

## Missing proteins in plant proteomic analysis

Recently, technical advances, especially in liquid chromatography (LC) and mass spectrometry (MS), have improved the sensitivity, coverage, reliability, and throughput of proteome analysis (Boersema et al., 2015). Novel proteomics methods, such as targeted proteomics (Marx, 2013), degradomics (Stoehr et al., 2013), structural proteomics (Walzthoeni et al., 2013), chemical proteomics (Rudolf et al., 2013), and microproteomics (Kasuga et al., 2017), are becoming essential tools for in-depth analyses of biological systems and phenomena, such as plant growth, development, and responses to stress factors.

The numbers of plant proteins detected using MS-based proteomics remains much lower than expected. For example, the improved maize reference genome contains 39,324 protein-coding genes, with an average of 3.3 transcripts per gene (Jiao et al., 2017), each of which may produce at least several different proteins. Moreover, additional proteins may be synthesized by proteolysis of other existing proteins. To date only 947 reviewed and 169,813 unreviewed maize protein entries have been collected in the UniProtKB (http://www.uniprot.org/uniprot/?query=organism:“maize”) (retrieved on Feb 6, 2018). Similarly, analysis of the UniProtKB entries for maize organelle proteins reveals few reviewed proteins compared with a large number of unreviewed entries (Supplementary Table 1). An important reason for this phenomenon is that maize proteomic data has not be curated and collected as the annotation of unreviewed protein entries. Definitely, numerous “missing (hidden) proteins” that are predicted at the transcript level remain unidentified at the protein level in plants.

While many factors contribute to missing proteins, one major cause is using inefficient protein extraction methods, especially for hydrophobic membrane proteins and low-abundant proteins (LAPs) (Thelen and Peck, 2007; Libault et al., 2017). Sample quality is critical for the coverage, reliability, and throughput of plant proteomic analysis, although advanced detection approaches (especially LC-MS/MS) can greatly enhance the sensitivity and reliability of protein identification. Here, in view of the current approaches and trends in plant proteomics, we highlight the importance of using multiple protein extraction methods to obtain a more complete picture of plant proteome. Moreover, to promote the identification of more “missing proteins,” we discuss the key aspects of protein extraction methods at the tissue, single-cell, and organelle levels.

## Extraction of total proteins for comparative proteomic analysis

Comparative proteomic analysis is mostly conducted using total proteins extracted from tissues, organs, or whole plants. Such approaches are effective to understand plant activities at the corresponding level, but suffer from a “dilution” effect that masks the unique biological properties of individual cells and cell-types (Libault et al., 2017).

A major challenge in plant proteomics is the effective and comprehensive extraction of proteins from plant tissues, due to the high dynamic range of plant proteins and the high levels of interfering substances (e.g., phenolics, lipids, organic acids, carbohydrates, terpenes, and pigments) (Wang et al., 2008). Therefore, for total proteins extraction from plant tissues, it is important to consider each of the following steps.

First, the extraction scale should be decided at an early stage. Plant tissues can be easily homogenized with quartz sand in the extraction buffer or pulverized with liquid N_2_ in a mortar. A small amount of plant materials (0.1–1.0 g fresh weight, depending on tissue type) is usually sufficient for proteomic analysis (Wu et al., 2014a).

Second, removal of interfering substances is necessary for preparing high-quality protein samples. To this purpose, two approaches are currently used: based on acetone/TCA precipitation and based on phenol extraction (Wang et al., 2008). Many pioneering works have contributed to the development, evaluation, and optimization of these approaches (Santoni et al., 2000; Giavalisco et al., 2003; Wang et al., 2003; Friso et al., 2004; Rose et al., 2004; Carpentier et al., 2005; Isaacson et al., 2006). Acetone/TCA precipitation method works well for almost plant tissues (Wang et al., 2008). Following acetone/TCA precipitation, organic-soluble substances are rinsed away, leaving proteins and other insoluble substances in the precipitate. Proteins are extracted using a buffer suitable for 2DE, iTRAQ, or LC-based separation. Phenol extraction method works by selectively extracting proteins from aqueous extracts during phase separation (Wu et al., 2014a). The profiles of the extracted proteome are highly dependent on the extraction buffers used (Chatterjee et al., 2012; Petriccione et al., 2013; Wu et al., 2014b). In addition, when using this approach one must also consider temperature (Wu et al., 2014b), pH (Sari et al., 2015), and extraction times (Feiz et al., 2006). Changing any of these parameters will affect the profile of the extracted proteome (e.g., Sari et al., 2014, 2015; Zhang et al., 2014). The success of the acetone/TCA precipitation and the phenol extraction approaches relies on the plant tissue being completely pulverized (Wu et al., 2014a).

Third, complex protein samples can be pre-fractionated to deplete high-abundance proteins, to enhance the detection of “missing” low-abundant proteins (LAPs). For example, the depletion of RuBisCO in leaves (Kim et al., 2013; Gupta and Kim, 2015) and of storage proteins in seeds (Xiong et al., 2014) and tubers (Wu et al., 2012; Kim et al., 2015; Lee et al., 2015; Gupta et al., 2016) significantly improved the separation and detection of LAPs.

Finally, each extraction method produces distinct protein complements. Therefore, integrating the application of different extraction methods will improve proteome coverage. Indeed, the importance of using multiple protein extraction methods to obtain comprehensive proteome coverage has been highlighted by several researchers (e.g., Karthikaichamy et al., 2017; Takác et al., 2017).

## Protein extraction for organelle proteomics

The low abundance of proteins in specific subcellular locations can result in their missing from tissue, organ, or whole plant protein samples (Libault et al., 2017). Therefore, the isolation of pure organelles allows for the analysis of LAPs that are specifically accumulated within them.

Using isolated organelles for protein extraction significantly reduces the complexity of the extracted proteome. This approach also enriches the LAP fraction in protein extracts, allowing for their improved separation and detection. Extensive proteomic studies of purified organelles, such as chloroplasts (Hall et al., 2011; Piro et al., 2015), nuclei (Sikorskaite et al., 2013), mitochondria (Lang et al., 2011; Salvato et al., 2014), and starch granules (Xing et al., 2016), have characterized a number of organelle proteins, and defined their localization information.

Previous cell biology and biochemistry studies have developed protocols for the isolation and purification of organelles including via homogenate filtration, differential centrifugation, and density gradients centrifugation (Table 1). The purity and integrity of extracted organelles can be tested by enzyme activity assay, light and electron microscopy, immunoblotting, and MS/MS identification. In contrast to the pulverization of plant tissue for total protein extraction, the extraction of organelle proteins requires gentle grinding to obtain pure and/or intact organelles before protein extraction.

**Table 1:** Examples of organelle isolation and subproteome analysis in plants.

**Materials**	**Organelles**	**Isolation protocols**	**Purity tests**	**Marker proteins**	**Protein extraction**	**Proteomic methods**	**Notes**	References^*^
*Posidonia oceanic* leaves	Chloroplast	Differential centrifugation, Percoll and sucrose gradient centrifugation	Enzyme activities, immunoblotting	Coproporphyrinogen III oxidase	Phenol extraction	SDS-PAGE, HPLC-Chip/MS	43 chloroplast proteins identified	Piro et al., 2015
Arabidopsis leaves	Chloroplast	Differential centrifugation, Percoll gradient centrifugation	Fluorescence microscopy, immunoblotting, enzyme activities	Fumarase, catalase	Stepwise extraction using Tris-HCl buffer, 2DE rehydration medium and 4% SDS solution	SDS-PAGE, LC/ MS/MS	690 proteins identified from purified chloroplasts	Kleffmann et al., 2004
Chickpea seeds	Chloroplast	Filtration, Percoll gradient centrifugation	Chlorophyll determination, immunoblotting, enzyme activities	LHCII, CPN 60, TOC 75, CoxII, V-ATPase	7M urea solution extraction	SDS PAGE, LC-MS/MS	2451 proteins identified	Lande et al., 2017
*Brassica napus* embryos	Plastid	Filtration with different size sieve	As above	Biotin carboxylase carrier protein	0.5 × SDS-PAGE buffer extraction	SDS-PAGE, Semi-continuous MudPIT MS/MS	Often being referred	Jain et al., 2008
*Medicago truncatula* roots	Plastid	Differential centrifugation, filtration, gradient centrifugation	Nitrite reductase activity, immunoblotting	Cytosolic UDP-Glucose pyrophosphatase	Acetone precipitation	SDS-PAGE, GeLC-MS/MS	266 proteins identified	Daher et al., 2010
Wheat kernels	Starch granule	CsCl gradient centrifugation	Electron microscopy	α-glucan phosphorylase	TCA/acetone precipitation and phenol extraction	2D-GE, peptide mass fingerprinting and LC-MS/MS	85 proteins identified	Bancel et al., 2010
Rice endosperm	Starch granule	Filtration with different size sieves	I_2_ staining, microscopy, immunoblotting	α-glucan phosphorylase	Phenol extraction	SDS-PAGE, LC-MS/MS	1157 proteins identified	Xing et al., 2016
Wheat florets	Mitochondrion	Differential centrifugation, filtration, Percoll gradient centrifugation	Enzyme activities, electron microscopy, 0.01 M Janus green B staining	Cytochrome c oxidase	2DE rehydration buffer extraction	2D-GE, LC-MS/MS	71 proteins identified	Wang et al., 2015
Potato tubers	Mitochondrion	AS above	Immunoblotting	Enolase, plastidic α-carboxytransferase, mitochondrial PDE1-α	SDS extraction	SDS-PAGE, GeLC-MS/MS	1060 proteins identified	Salvato et al., 2014
Arabidopsis suspension cells	Vacuole	Differential centrifugation, Percoll and sucrose gradient centrifugation	Enzyme activities, immunoblotting	V-type H^+^-ATPase	SDS extraction	SDS-PAGE, LC-MS/MS	163 proteins identified; often being referred	Shimaoka et al., 2004
Tobacco, potato, apple leaves	Nucleus	Filtration, differential centrifugation, Percoll and sucrose gradient centrifugation	DAPI staining, microscopy, immunoblotting	OsRH36-GFP	TRizol reagent extraction	SDS-PAGE	none	Sikorskaite et al., 2013
Arabidopsis suspension cells	Golgi apparatus	c-Myc tag; iodixanol density ultra-centrifugation	Immunoblotting	Gtl6/At2g29900	TCA/acetone precipitation	LC-IMS-MS	102 Golgi-localized proteins identified	Nikolovski et al., 2014
AS above	Golgi apparatus	Sucrose gradient centrifugation	Immunoblotting, electron microscopy, enzyme activities	NDPase	FFE buffers extraction	SDS-PAGE, LC-MS/MS	371 proteins identified	Parsons et al., 2012
Castor seeds	Endoplasmic reticulum	Differential centrifugation, filtration, sucrose gradient centrifugation	MS/MS identification	Oleate-12-hydroxylase	DOC-TCA precipitation	SDS-PAGE, 2D-GE, MALDI-TOF-MS, Q-TOF MS/MS	300 proteins identified, often being referred	Maltman et al., 2002
Arabidopsis leaves	Peroxisome	AS above	Enzyme activities, MS/MS identification	Hydroxypyruvate reductase	2DE rehydration buffer extraction	2D-GE, LC-MS/MS	Often referred	Reumann et al., 2007
Arabidopsis hypocotyls	Cell wall	Filtration, differential centrifugation	LC-MS/MS identification	N-glycoprotein	Salt/SDS buffer extraction	2D-GE, LC-MS/MS	Often referred	Feiz et al., 2006
Alfalfa stems	Cell wall	Low-salt/gradient centrifugation	As above	N-glycoprotein	EGTA/LiCl solution extraction and methanol/chloroform precipitation	SDS-PAGE, LC-MS/MS	245 proteins identified	Verdonk et al., 2012

*These studies were selected because the isolation protocols allowed for high-purity of specific organelles and the large number of organelle proteins identified by subproteomics analysis*.

Some organelles are relatively easy to isolate from others, especially those with storage functions (e.g., lipid-bodies and starch granules) and large organelles with membranous structures (e.g., chloroplasts and mitochondria). Novel methods are constantly being developed to isolate difficult organelles for subproteomic analysis, e.g., a combination of density centrifugation and surface charge separation techniques to isolate pure Golgi membranes (Parsons et al., 2012), Percoll gradient centrifugation followed by sucrose gradient centrifugation to isolate peroxisomes (Reumann and Singhal, 2014), and a simple density gradient (ultra-)centrifugation protocol to isolate intact vacuoles (Ohnishi et al., 2018) from Arabidopsis suspension cultured cells.

Once organelles are isolated, standard protein extraction approaches can be used. The composition of protein extraction buffers can be altered to suit the properties of target proteins (e.g., solubility, hydrophobicity or hydrophilicity, p*I*, and the degree associated with membranes). Importantly, for organelles with membrane structures, the membranes need to be broken by grinding, sonication, enzyme digestion, or detergent lysis to release soluble proteins (Lang et al., 2011; Piro et al., 2015).

For the organelles with complex structures, the separate extraction of proteins from each suborganelle fraction enables producing more detailed subproteome profiles. For example, subproteomic analysis involving the isolation of Arabidopsis chloroplasts as stroma, thylakoid membrane, and lumen fractions (Hall et al., 2011) and the separate isolation of inner and outer mitochondrial membrane fractions (Duncan et al., 2011; Schikowsky et al., 2018) have also provided information about the specific localization of proteins within the organelles.

Finally, compared with proteins in intracellular compartments, a major technical challenge in extracting cell wall proteins (CWPs) is the preparation of a pure cell wall sample. This is particularly challenging because substantial amounts of intracellular proteins inevitably associate with the cell wall during the process of tissue or cell homogenization (Rose and Lee, 2010). Cell wall isolation methods have been optimized (Feiz et al., 2006; Zhang et al., 2011; Printz et al., 2015) and, in general, the cell wall proteome consists of *sensu stricto* CWPs, apoplast proteins, secreted proteins, and xylem sap proteins (Wu et al., 2018). Most loosely bound cell wall proteins can be dissolved using a low ionic strength solution, while strongly bound cell wall proteins are resistant to salt-extraction (Jamet et al., 2008). Besides, the extraction and proteomic analysis of apoplast proteins, secreted proteins and xylem sap proteins (Soares et al., 2007; Kim et al., 2014) have made important achievements.

## Protein extraction for single cell-level proteomics

Another reason that proteins can be missing from plant proteomic analysis is that some LAPs (e.g., transcription and regulatory factors) accumulate in specialized cell or tissue types and at specific development stages (Dubos et al., 2010). In entire organ, or whole plant analyses, the presence of these proteins is often masked by that of high-abundance proteins. Therefore, single cell level proteomics or microproteomics will minimize the cellular complexity of the analyzed sample (Libault et al., 2017). However, sample preparation and protein extraction techniques for microproteomic analysis of plant tissues remain challenging.

Microproteomic techniques rely on accurate and precise sample collection, preparation, excision, and protein extraction (Feist and Hummon, 2015). Laser capture microdissection (LCM) is a promising method for cell level sampling. LCM allows cell types of interest to be isolated of from a fixed sample under direct microscopic visualization with the assistance of a laser beam. LCM has been successfully used in the proteomic analysis of Arabidopsis (Schad et al., 2005), maize (Dembinsky et al., 2007), barley (Kaspar et al., 2010), and tomato (Zhu et al., 2016). The best example of the application of LCM, combined with pressure catapulting, was to isolate the nucellar projection and endosperm transfer cells of an developing barley grain at 8 days post-flowering. The protein extracts were analyzed by nanoUPLC separation combined with ESI-Q-TOF MS, which successfully identified 137 and 44 proteins in nucellar projection and endosperm transfer cells, respectively (Kaspar et al., 2010). In addition, a method of mechanical separation of leaf epidermal, vascular, and mesophyll tissues has been developed in Arabidopsis (Falter et al., 2015), tomato, and cassava (Svozil et al., 2016), and the separated tissue samples can be used for quantitative LCM-assisted microproteomic analysis.

It takes a lot of time and effort to obtain sufficient numbers of cells from limited samples using LCM. Therefore, it is necessary to develop micro-scale protein extraction methods, compatible with decreased sample size (100 μg and less), to use in parallel with this approach to generate high-quality MS data for “missing” LAPs.

## Concluding remarks

Many “missing” proteins have not been proven at the protein level. Therefore, we have emphasized the importance of optimization of protein extraction methods to enhance the detection of the missing proteins in plant proteomics. Surely, MS-based proteomics alone is not sufficient to explore and identify all missing proteins. Integrated multi-omics approaches will facilitate the identification of many of the missing proteins (Chang et al., 2014).

It is necessary to note that the aim of the Opinion article is not to review previous studies, but to highlight the importance of developing novel approaches to establish plant proteomes. Special attention should always be paid to developing quantitative, reproducible, and comparable methodologies for plant proteomics. Particularly, suitable protein extraction methods integrating with isolation techniques for organelles, specific cells and tissues will greatly enhance plant proteomic analysis and allow to identify more “missing proteins.” Good protein extraction makes for a good proteome.

## Author contributions

All authors contributed to the writing of the manuscript. LN and WW revised the manuscript.

### Conflict of interest statement

The authors declare that the research was conducted in the absence of any commercial or financial relationships that could be construed as a potential conflict of interest.
