# Supplementary material for: Protein Extraction Methods Shape Much of the Extracted Proteomes
Source: Front Plant Sci. 2018 Jun 12;9:802. doi: 10.3389/fpls.2018.00802 (PMC6005817; doi:10.3389/fpls.2018.00802)
Supplement: Supplementary file 1 [file Table_1.DOCX]

**Supplementary Table 1 |** The number of maize proteins located or predicated in different organelles.

| **Organelles** | **Reviewed entries** | **Unreviewed entries** |
| --- | --- | --- |
| Chloroplast | 197 | 1398 |
| Plasma membrane | 64 | 1019 |
| Nucleus | 61 | 1781 |
| Cytoskeleton | 39 | 612 |
| Mitochondrion | 39 | 153 |
| Cell wall | 32 | 1155 |
| Endoplasmic reticulum | 30 | 1157 |
| Vacuole | 24 | 293 |
| Golgi apparatus | 5 | 726 |
| Peroxisome | 5 | 451 |
| Starch granule | 1 | 51 |
| Lysosome | 1 | 4 |

*Note: The data was* *retrieved from the UniProtKB (*[*http://www.uniprot.org/*](http://www.uniprot.org/)*) on Feb 6, 2018. Reviewed entries mean that the existence of these proteins has been proved at the protein level, while unreviewed entries means that the existence of these proteins were based on genome and transcriptome data.*
